# Purpura Fulminans Secondary to Haemophilus influenzae in an Infant

**DOI:** 10.7759/cureus.83313

**Published:** 2025-05-01

**Authors:** Amy Ko, Shanzay Mohammad, Arjun Chandran, Summer D Donovan, Niveditha Balakumar

**Affiliations:** 1 Pediatrics, Baylor College of Medicine, Houston, USA; 2 Pediatrics, Christus Children's Hospital, San Antonio, USA; 3 Pediatric Critical Care, Baylor College of Medicine, Houston, USA; 4 Pediatric Critical Care, Christus Children's Hospital, San Antonio, USA; 5 Pediatric Infectious Diseases, Baylor College of Medicine, Houston, USA; 6 Pediatric Infectious Diseases, Christus Children's Hospital, San Antonio, USA

**Keywords:** acute infectious purpura fulminans, haemophilus influenzae, haemophilus influenzae serotype b conjugate vaccine, plasma exchange therapy, severe sepsis

## Abstract

Purpura fulminans (PF) is a hematological emergency that requires timely diagnosis for effective management. In this report, we discuss the case of a five-month-old previously healthy male who presented to the emergency department due to sudden-onset lethargy and rash in the setting of intermittent fevers, emesis, and diarrhea. Shortly after admission, the patient was noted to have rapidly progressive PF and was intubated due to impeding respiratory failure and hemodynamic instability requiring the institution of vasopressors and steroids for catecholamine-resistant, refractory septic shock. He was initially managed with a course of broad-spectrum antimicrobials, including ceftriaxone, doxycycline, and vancomycin, while awaiting the results from his blood and urine cultures. Unfortunately, our patient progressed to multi-organ failure and disseminated intravascular coagulation requiring advanced organ support, including the need for continuous renal replacement therapy. He was transferred to another facility for evaluation and continued management of his necrotic and gangrenous tissues, where he ultimately died after experiencing septic shock in the setting of bacteremia. Although blood and urine cultures were negative, his microbial cell-free DNA sequencing (Karius test) was positive for *Haemophilus influenzae*, the likely causal pathogen. *Haemophilus influenzae* is a Gram-negative coccobacillus that is commonly associated with invasive bacterial infections in the pediatric population. With the widespread implementation and utilization of the *H. influenzae* type B vaccine as part of routine vaccinations, the incidence of severe illness is rare. Only a handful of cases of* H. influenzae *causing PF are reported in the literature, and it is uncommon in the pediatric population, emphasizing the need to report and understand such occurrences. This case specifically highlights the importance of early recognition and timely management of PF, a rapidly degenerative clinical condition, in the setting of severe sepsis and multi-system organ failure secondary to an unlikely pathogen.

## Introduction

Purpura fulminans (PF) is an acute dermatologic finding that indicates hematologic compromise and coagulopathy. Known for its trademark purpuric skin lesions, this clinical finding acts as a harbinger of the consequent necrosis and, if diagnosis or treatment is delayed, impending organ failure [1]. PF is often associated with a very high mortality rate, ranging from 40% to 50%, with patients often perishing due to rampant multi-system thrombosis rather than septic shock [1-3]. Commonly, the etiologies of PF are divided into three categories: infectious or acute septic, hereditary or neonatal, and post-infectious or idiopathic [4]. In acute septic PF, coagulopathy has been found to be associated with the release of cytokines secondary to the initiation of the coagulation cascade. Hereditary PF has been found to be associated with a congenital protein C deficiency, protein S deficiency, and antithrombin III deficiency [5]. Lastly, post-infectious PF is believed to be caused by the acquired deficiency of PS due to the accumulation of anti-PS autoantibodies, with onset being the week following either a viral or bacterial infection [4].

With the introduction of the *H. influenzae* type B (Hib) vaccine in the United States in 1985, there has been a decline in invasive infections caused by *H. influenzae* [2]. In the past 40 years, there have been a total of 16 pediatric and three adult cases of *H. influenzae* related to PF worldwide [2,3,6]. With the introduction of the Hib vaccine, reported pediatric cases of Hib-related PF in the United States have been very rare, with one case in 1985 and another between 1986 and 1998 [7,8]. Interestingly, with the implementation of the Hib vaccine, there has been an increase in the incidence of non-typeable *H. influenzae* infections [6,9]. As Hib-related infections have been fewer in number due to routine immunizations, there has been some speculation that virulent tendencies may emerge from other *H. influenzae *serotypes. In Utah, for example, there were five notable cases of severe disease related to *H. influenzae *type A, four with bacteremia and meningitis and one with PF [10]. These findings highlight the potential genesis of virulence in previously benign serotypes, a humbling reminder that though vaccinations provide an excellent means of defense against certain diseases, microorganisms maintain the innate ability to evolve and alter their means of offense.

The objective of this case report is to emphasize the importance of expeditious diagnosis of septic shock in the setting of PF by familiarizing providers with associated clinical findings and to initiate effective treatment to avert physiological deterioration and prevent fatal comorbidities. Our aim is to also review the variance of virulence in *H. influenzae* in recent literature. Given its close association with precipitous physiologic decline, clinicians should be familiar with and remain vigilant of PF so that they are prepared for prompt action if ever presented with this finding.

## Case presentation

**Figure 1:**
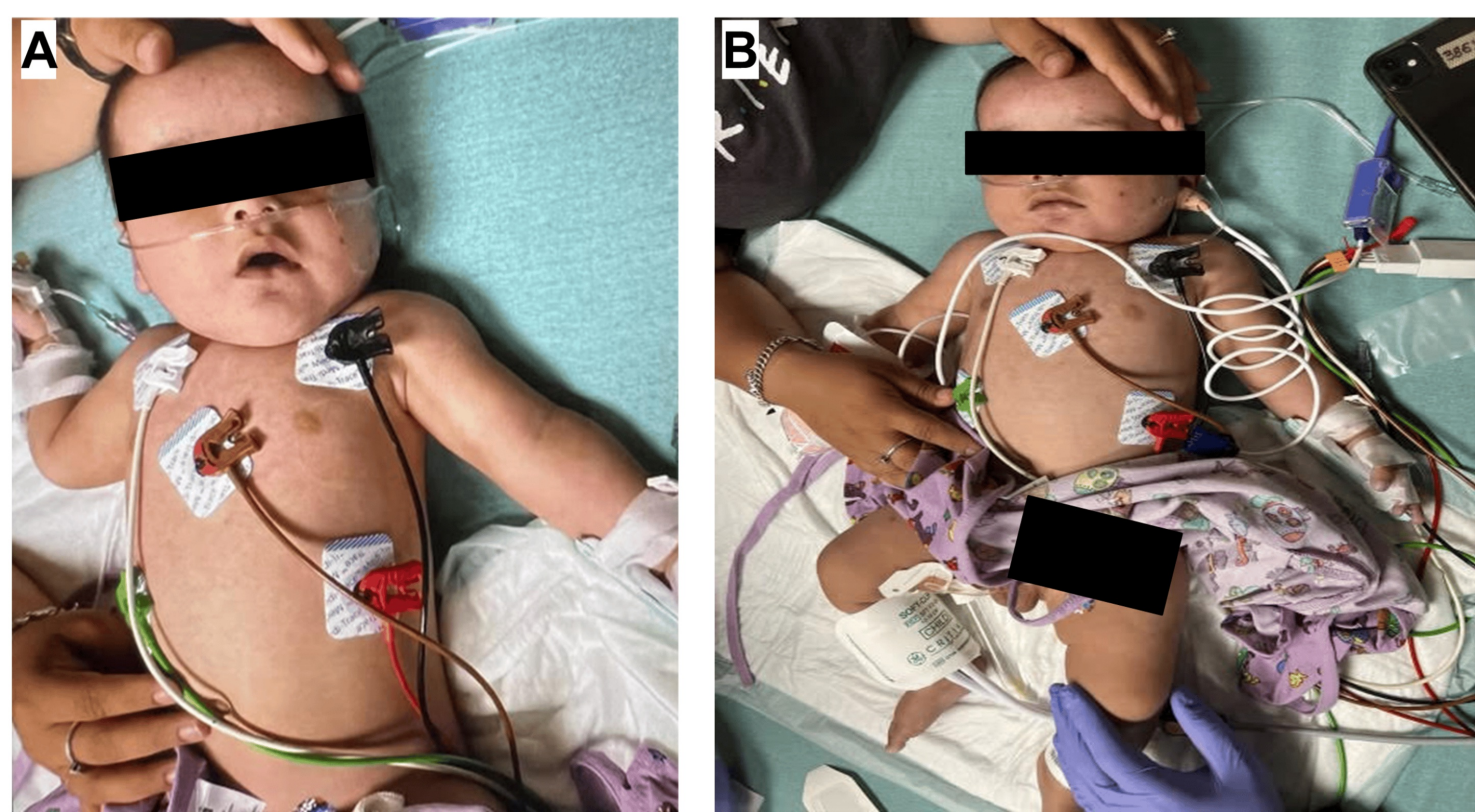
(A, B) Patient at outside emergency department on hospital day 1 noted to be lethargic with mild petechiae on the face.

A previously healthy five-month-old partially immunized male, who had received his up to two-month vaccines and missed his four-month vaccines, presented to an outside emergency department (ED) with sudden-onset lethargy and a two-day history of intermittent fevers and five days of vomiting and diarrhea. Prior to going to the ED, he was seen at an urgent care clinic on day three of symptoms of mild congestion and subjective fevers, where COVID-19 and flu tests were negative, and the family was advised supportive care. Over the following two days, the patient developed fevers, increasing fussiness, poor oral intake, and the beginnings of a purple hue to his skin, prompting his family to seek emergency care. His physical examination on ED admission revealed a poorly responsive five-month-old male with a purple-gray skin tone (Figure 1).

Initial lab work was significant for thrombocytopenia, neutropenia, and profound lactic acidosis, and during his hospitalization, these labs were trended to monitor his clinical status (Table 1).

**Table 1:** Relevant lab results from initial and subsequent days of hospitalization. ↑↑ indicates "critically high" and ↓↓ indicates "critically low" WBC, white blood cells; ANC, absolute neutrophil count; PLT, platelets; BUN, blood urea nitrogen; Cr, creatinine; CRP, C-reactive protein; PT, prothrombin time; INR, international normalized ratio; PTT, partial thromboplastin time; BNP, B-type natriuretic peptide

	Day 1	Day 3	Day 5	Day 7	Day 10
WBC	1.9↓↓	3↓	10.2	31.5↑↑	32.3↑↑
ANC	170↓↓	1500	7240	25200↑	28100↑
PLT	84↓	110↓	<10↓↓	38↓↓	37↓↓
BUN	11	17	11	33↑	31↑
Cr	0.8	0.5	0.4	0.4	0.3
Albumin	1.6↓	2.7↓	2.6↓	2.3↓	2.4↓
CRP	17.23↑	18.16↑	16.8↑	15.15↑	6.97↑
pH	6.943↓↓	7.365	7.354	7.42	7.497
Lactate	10.88↑↑	14.37↑↑	8.2↑↑	6.08↑↑	2.61
PT	36.9↑	16.3↑	15.6↑	15.3↑	13.8
INR	3.58	1.27	1.2	1.14	1
PTT	129.6↑↑	37.76↑	31.4	31.5	31.8
Fibrinogen	92↓↓	320	375	424	494↑
D-dimer	7.64↑				
Functional protein C	21↓				
Procalcitonin	495.44↑↑	125.41↑	18.66↑		
BNP	1666↑↑	500↑			
Troponin	0.36↑↑				

**Figure 2:**
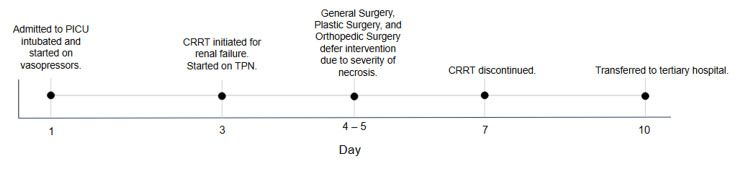
Timeline of key clinical events from presentation to transfer to outside tertiary hospital. CRRT, continuous renal replacement therapy; TPN, total parenteral nutrition; PICU, pediatric intensive care unit

The patient was intubated for altered mental status and worsening hemodynamic instability. He was transferred to a tertiary pediatric intensive care unit (PICU), and within about one hour of arrival to the PICU, he developed refractory shock, which was poorly responsive to fluid resuscitation, and required escalating doses of vasoactive infusions, with further need for stress-dose steroids due to concerns for catecholamine-resistant shock. A visual timeline of the patient's clinical progression is provided in Figure 2.

**Figure 3:**
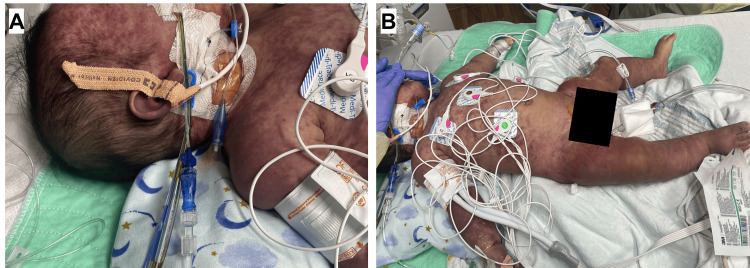
(A, B) Patient within two hours of arrival to PICU on hospital day one with worsening petechiae and purpura across the face, chest, trunk, and extremities.

The rapidity of progression, along with the drastic development of his rash into purpuric lesions with petechiae across his face, chest, trunk, and extremities, solidified the diagnosis of PF (Figure 3).

Empiric antibiotics included doxycycline, vancomycin, and ceftriaxone. His labs continued to show ongoing disseminated intravascular coagulation (DIC), pancytopenia, and severe metabolic acidosis. On hospital day 3, he progressed to renal failure and developed anuria, which prompted the decision to initiate continuous renal replacement therapy (CRRT). Therapeutic plasma exchange was done as adjunct supportive therapy. Vasoactive infusions were slowly weaned as his hemodynamics improved, with close monitoring to ensure that his blood pressure remained within the age-appropriate range. Though blood cultures were negative, next-generation sequencing of microbial cell-free DNA (Karius) testing identified the offending pathogen as *H. influenzae* with a microbial load of 240,000 molecules per microliter.

**Figure 4:**
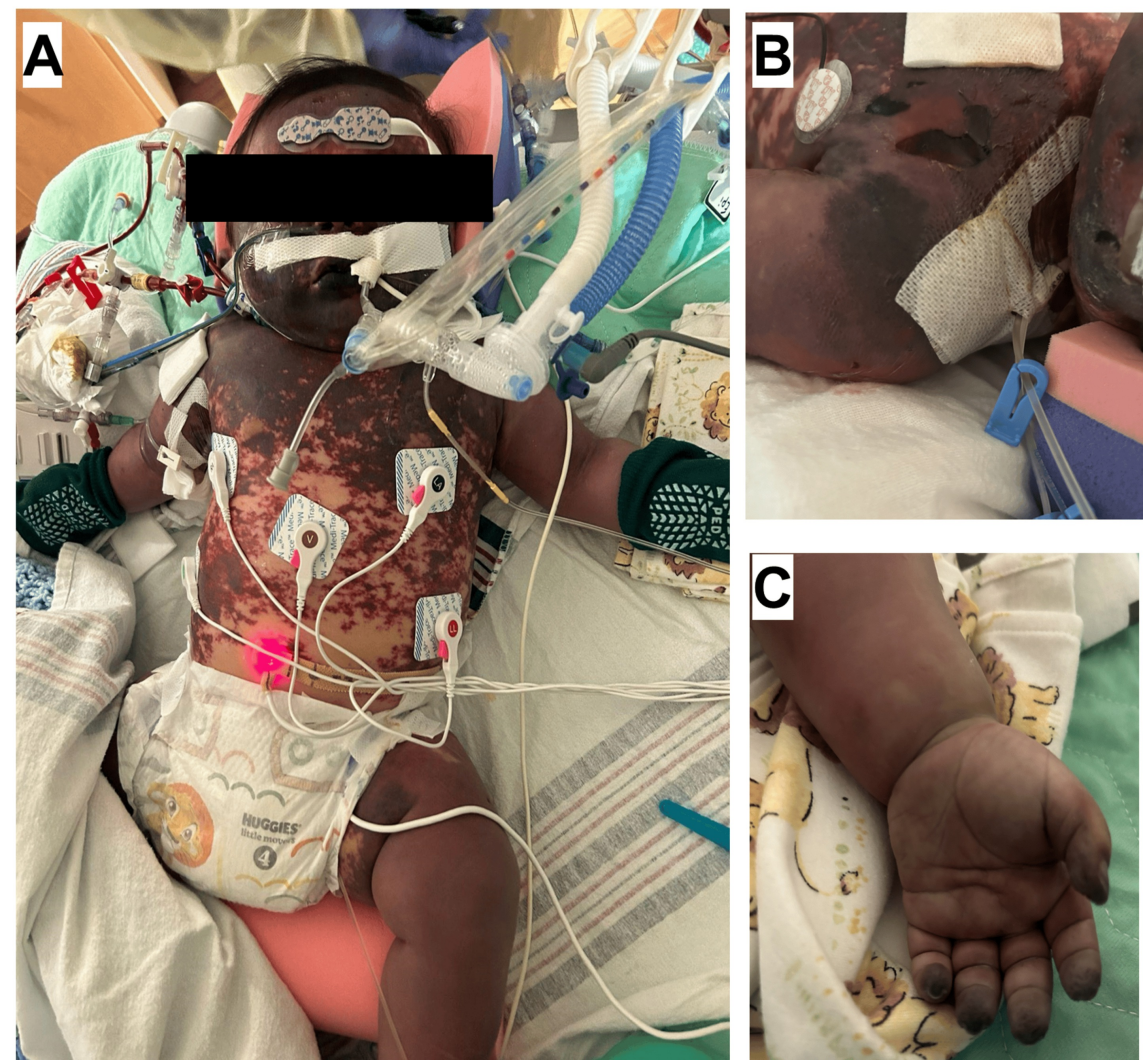
(A) Progressively worsening purpura fulminans on hospital day 5. (B) Skin breakdown was noted on the face, shoulders, and trunk. (C) Necrosis was also noted on the fingers.

Our patient was managed by a multidisciplinary team comprised of plastic surgery, general surgery, orthopedic surgery, and wound care. In addition to routine care, nitroglycerin paste was applied to distal extremities for vasodilation, though with minimal improvement (Figure 4). During his PICU hospitalization, the integrity of his skin continued to show signs of significant breakdown, and his appendages showed signs of necrotic damage (Figures 4B, 4C).

Our patient was evaluated for the development of compartment syndrome and the need for potential tissue debridement but did not require any emergent surgical interventions or fasciotomies. On hospital day 10, he was transferred to another tertiary hospital that specialized in multi-specialty wound care with the ability to provide a more comprehensive evaluation of his gangrenous and necrotic tissue. Of note, vasoactive infusions and CRRT were discontinued prior to transfer with improvement in his renal function, with vasoactive agents being discontinued on hospital day 6 and CRRT being discontinued on hospital day 7. Before his transfer, he also received 19 units of fresh frozen plasma, 8 units of packed red blood cells, and 13 units of platelets.

Given the patient's physical examination findings of progressively worsening PF, septic shock, and DIC, the initial consideration was meningococcal or pneumococcal sepsis. However, a broad differential of infectious etiologies was considered. His blood and urine cultures were negative. An endotracheal tube culture collected on hospital day 7 grew *Pseudomonas aeruginosa*. Infectious workup for malaria, rickettsia, enterovirus, and dengue virus were all negative. At the tertiary hospital, the patient underwent a tracheostomy due to significant facial sloughing that compromised the ability to secure the endotracheal tube. He was also tested and evaluated for hypercoagulable states, including factor V Leiden, prothrombin G20210A, and protein S deficiency, all of which were negative. ADAMTS13 (a disintegrin and metalloproteinase with thrombospondin type 1 motif, 13) was normal, ruling out thrombotic thrombocytopenic purpura. Despite initial improvement, he developed septic shock due to *Pseudomonas* bacteremia after transfer to the tertiary hospital. The decision was made to withdraw life-sustaining measures, and he died later that day.

## Discussion

In the United States, there has been a notable decrease in pediatric cases of severe infections related to *H. influenzae* following the introduction of the Hib vaccine [2,3,6]. In the three reported adult cases of PF secondary to invasive *H. influenzae*, all patients experienced complications such as limb amputations, secondary infections, and/or multi-organ failure, indicating that bacteremia due to *H. influenzae* in the setting of PF may be associated with increased risk of morbidity and potentially mortality [2,3,11]. Notably, there has been only a single case of non-typeable *H. influenzae *causing PF in the adult population [3]. There have been reported cases of *H. influenzae* being the causal organism for necrotizing fasciitis in the adult population, with one intriguing case observed in the setting of COVID-19 pneumonia [12].

PF results in ischemic necrosis of the extremities secondary to the disruption of hemostasis, leading to the formation of obstructive microthrombi [1]. The fatal sequelae arise from dysregulation of coagulation, with patients often succumbing to hematologic complications rather than the initial insult itself [1]. Traditionally, the causes of PF have been divided into three broad categories: infectious or acute septic, hereditary or neonatal, and post-infectious or idiopathic [4]. The infectious etiologies can be further delineated into bacterial, such as *N. meningitidis*, *S. pneumoniae*, and *H. influenzae*, as well as viral, such as varicella, rubella, and measles [2,4]. Hereditary or neonatal etiologies include protein C deficiency, protein S deficiency, and antithrombin III deficiency, with congenital protein C deficiency being the most common [5]. In congenital protein C deficiency, an autosomal recessive thrombophilia, PF is considered one of the initial clinical presentations and typically presents within the first 24 to 48 hours of life [5]. Finally, the post-infectious or idiopathic category occurs within 7 to 10 days following a bacterial or viral insult, with the pathophysiology believed to result from the formation of anti-protein S autoantibodies [4].

Given the rapidly progressive nature and risk of hematological compromise, timely diagnosis and management of infectious PF are essential. The mainstay of treatment in the early stages includes providing adequate fluid resuscitation and initiating empirical broad-spectrum antibiotics to cover against the most common bacterial agents, such as N. meningitidis and S. pneumoniae [1,4]. DIC may require frequent blood product transfusions, such as fresh frozen plasma and cryoprecipitate [1,4]. The severe tissue necrosis resulting from compromised perfusion and vascular thrombosis also calls for extensive debridement and skin grafting of the affected areas, making prompt engagement of a multidisciplinary team, including experts in burn care, crucial [1]. There have been some case reports describing the use of hyperbaric oxygen to improve oxygenation in tissues and prevent further necrosis; however, there are insufficient prospective data to determine whether this treatment provides long-term benefits [2,4,9].

Plasmapheresis, an adjunctive therapy used in our patient, has yet to show decisive evidence of reducing mortality in cases of septic shock. The high rates of mortality and morbidity in patients with septic shock and subsequent PF is believed to result from the multi-organ failure caused by an uninhibited cytokine storm activated by the triggering pathogen. Plasmapheresis serves as a means of removing noxious mediators from circulation; thus, the rationale of utilizing plasmapheresis in this patient population is to neutralize the detrimental effects of the pervasive inflammatory response [13].

Although plasmapheresis has been utilized in this population for decades, studies in adults have reported conflicting results regarding its efficacy in the setting of sepsis. However, a randomized controlled trial including septic patients who received plasmapheresis demonstrated a significantly increased rate of survival [14]. A meta-analysis involving patients with sepsis and multi-organ dysfunction syndrome also found that adjunct therapeutic plasma exchange was associated with a decreased risk of short-term mortality [15]. While some cases suggest that plasmapheresis may reduce pediatric mortality, no large-scale randomized studies have definitively proven this [16-18]. In fact, one systematic review found that though plasmapheresis was associated with reduced mortality in the adult population, the same was not true for pediatric patients [19]. The American Society for Apheresis recommends plasmapheresis as an experimental treatment, emphasizing the need for further in-depth studies on the long-term efficacy of plasmapheresis in this population [20].

## Conclusions

Given the catastrophic physiological effects that can transpire following PF, it is imperative to focus on both preventive measures and early and targeted treatment. Though rates of complications due to invasive *H. influenzae* infections have decreased since the introduction of the Hib vaccine, our case highlights that *H. influenzae*, including non-type B strains, can cause significant morbidity and mortality. Therefore, early recognition and familiarity with the primary clinical signs and causes of PF in the setting of DIC and septic shock are critical for initiating prompt and effective management to mitigate the destructive course of the condition and prevent fatalities.
